# Multi-drug resistant *Klebsiella pneumoniae* strains circulating in hospital setting: whole-genome sequencing and Bayesian phylogenetic analysis for outbreak investigations

**DOI:** 10.1038/s41598-017-03581-4

**Published:** 2017-06-14

**Authors:** Eleonora Cella, Massimo Ciccozzi, Alessandra Lo Presti, Marta Fogolari, Taj Azarian, Mattia Prosperi, Marco Salemi, Michele Equestre, Francesca Antonelli, Alessia Conti, Marina De Cesaris, Silvia Spoto, Raffaele Antonelli Incalzi, Roberto Coppola, Giordano Dicuonzo, Silvia Angeletti

**Affiliations:** 1 Department of Infectious, Parasitic and Immune-Mediated Diseases, Istituto Superiore di Sanità, Rome, Italy; 2 Department of Public Health and Infectious Diseases, Sapienza University of Rome, Rome, Italy; 3 Unit of Clinical Pathology and Microbiology, University Campus Bio-Medico of Rome, Rome, Italy; 4 Department of Epidemiology, Center for Communicable Disease Dynamics, Harvard’s T.H. Chan School of Public Health, Boston, MA USA; 5 Department of Epidemiology, University of Florida, Gainesville, FL USA; 6 Department of Pathology, Immunology, and Laboratory Medicine, Emerging Pathogens Institute, University of Florida, Gainesville, FL USA; 7 Department of Cell Biology and Neurosciences, Istituto Superiore di Sanità, Rome, Italy; 8 Internal Medicine Department, University Hospital Campus Bio-Medico, Rome, Italy; 9 Unit of Geriatrics, Department of Medicine, University Campus Bio-Medico of Rome, Rome, Italy; 10 Department of Surgery, University Campus Bio-Medico of Rome, Rome, Italy

## Abstract

Carbapenems resistant *Enterobacteriaceae* infections are increasing worldwide representing an emerging public health problem. The application of phylogenetic and phylodynamic analyses to bacterial whole genome sequencing (WGS) data have become essential in the epidemiological surveillance of multi-drug resistant nosocomial pathogens. Between January 2012 and February 2013, twenty-one multi-drug resistant *K*. *pneumoniae* strains, were collected from patients hospitalized among different wards of the University Hospital Campus Bio-Medico. Epidemiological contact tracing of patients and Bayesian phylogenetic analysis of bacterial WGS data were used to investigate the evolution and spatial dispersion of *K*. *pneumoniae* in support of hospital infection control. The epidemic curve of incident *K*. *pneumoniae* cases showed a bimodal distribution of cases with two peaks separated by 46 days between November 2012 and January 2013. The time-scaled phylogeny suggested that *K*. *pneumoniae* strains isolated during the study period may have been introduced into the hospital setting as early as 2007. Moreover, the phylogeny showed two different epidemic introductions in 2008 and 2009. Bayesian genomic epidemiology is a powerful tool that promises to improve the surveillance and control of multi-drug resistant pathogens in an effort to develop effective infection prevention in healthcare settings or constant strains reintroduction.

## Introduction

Carbapenems resistant *Enterobacteriaceae* (CRE) infections are increasing worldwide. In Italy, sporadic cases or outbreaks caused by CRE have been reported since the early 2000s. Among CRE, the increase of carbapenem resistant *Klebsiella pneumoniae* strains since 2010 was of great concern as documented by the EARS-NET surveillance system^1, 2^.

Carbapenems resistance in *Klebsiella pneumoniae strains*, often results from the presence of plasmid-encoded *K*. *pneumoniae* carbapenemase (KPC)^2–4^, is one of the leading causes of hospital-acquired infections (HAIs), characterized by high rate of morbidity and mortality^3, 4^.

Despite this emerging health threat, little is known about the genetic diversity of *K*. *pneumoniae* strains circulating in hospital settings. Antimicrobial susceptibility profiles or molecular genotyping methods such as pulsed field gel electrophoresis (PFGE) or multilocus sequence typing (MLST) have largely been used to assess the relatedness among bacterial isolates. Since these methods, even providing information about the large-scale population structure of bacterial species, lack the discriminatory resolution to investigate epidemics on finite geographical or temporal scales^4, 5^, next-generation sequencing (NGS) has been applied at this aim. For example, whole genome sequencing was used to study a hospital outbreak of multidrug-resistant *Acinetobacter baumannii* in England combining WGS information with classical epidemiological data. By these means, the transmission events were reconstructed and adequate measure for hospital infection control improved^6^. Among patients undergoing endoscopic retrograde cholangiopancreatography (ERCP), patients have investigated infections by KPC K. pneumoniae and endoscope isolates comparison using WGS. By phylogenetic core SNPs analyses, the genetic relatedness of isolates obtained from endoscopes and patients was demonstrated^7^. In addition, analyses of bacterical WGS data has also been applied to methicillin resistant *S*. *aureus* (MRSA) nosocomial infections demonstrating improved discriminatory power compared to other typing techniques, such as Multi-Locus Sequence Typing (MLST), even in this genetically homogenous group^8, 9^. Roch *et al*. performed a prospective study applying WGS to all bacterial isolates obtained from a tertiary care hospital’s intensive care units and demonstrated that the genomic surveillance of clinical isolates provides a useful tool to highlight important differences among bacterial strains encouraging the introduction of microbial genome sequencing into routine clinical care^10^.

Phylogenetic and phylodynamic tools applied to bacterial whole genome and core SNPs analysis have become an essential component in the epidemiological surveillance of multi-drug resistant (MDR) pathogens to discern outbreak from non-outbreak strains in both community and hospital settings^11, 12^.

The combination of phylogenetic and evolutionary analyses, based on genome-wide SNPs, with clinical and epidemiological data represents a powerful tool to infer the origin and the spread of *K*. *pneumoniae* nosocomial strains. Isolates temporally and spatial related could clarify the epidemiological transmission as so as the eventual reservoir in the hospital setting supporting the epidemiological surveillance and infections control strategies.

In this study, *K*. *pneumoniae* KPC strains circulating within different wards of the University Hospital Campus Bio-Medico were collected and WGS applied. Classical epidemiological and Bayesian phylogenetic analysis were combined to evaluate transmission dynamics and evolutionary history of *K*. *pneumoniae* KPC in support of hospital infection control prevention efforts.

## Results

### Study population and epidemiologic investigation

**Figure 1:**
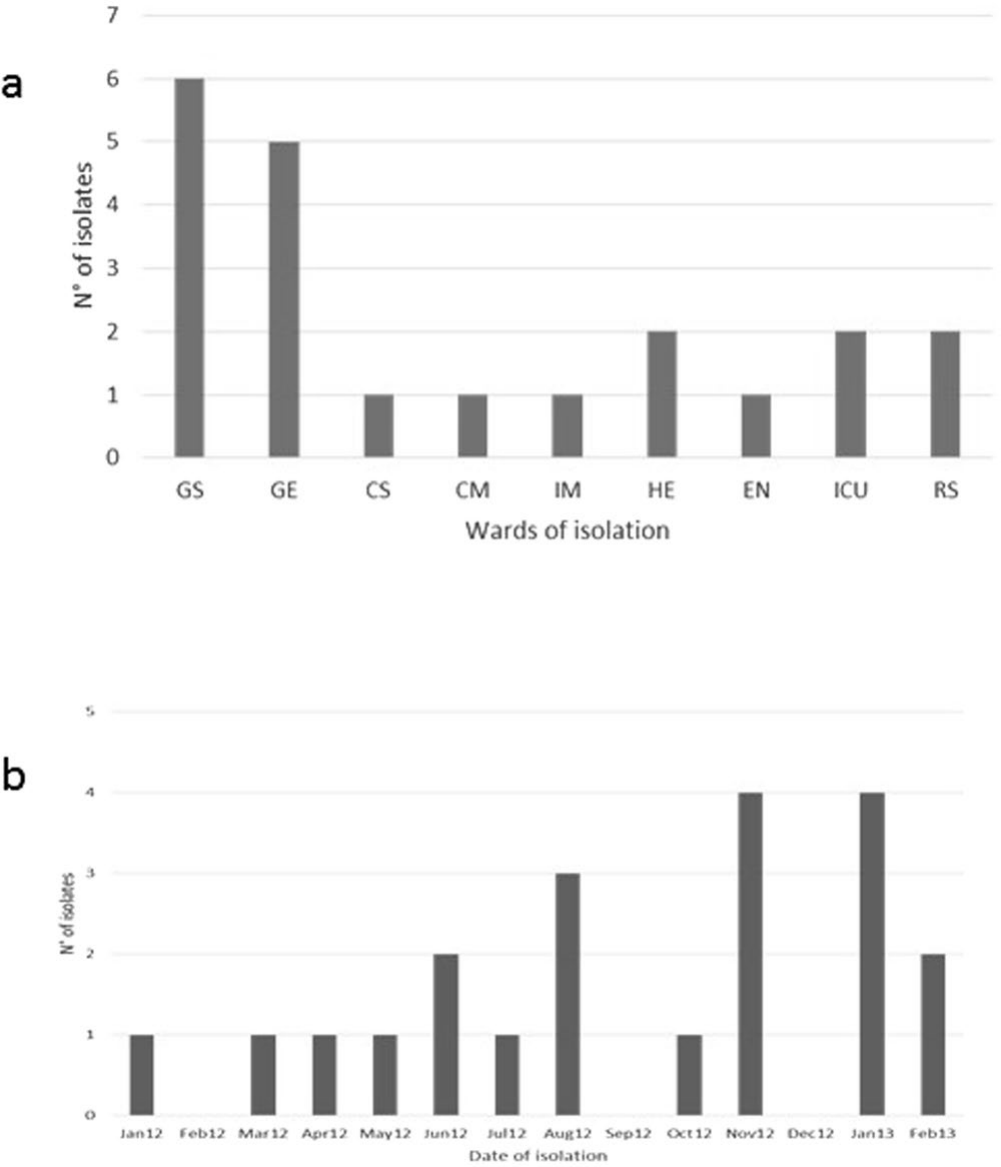
Epidemic curves based on the number of *Klebsiella pneumoniae* isolated in for each ward (**a**) and in the temporal frame of the study (**b**).

*Klebsiella pneumoniae* MDR and KPC strains included in the study were isolated from 21 patients distributed in different wards of the hospital as in Fig. 1a. Isolates were collected more frequently from patients admitted in the general surgery and geriatric wards (Fig. 1a).

**Figure 2:**
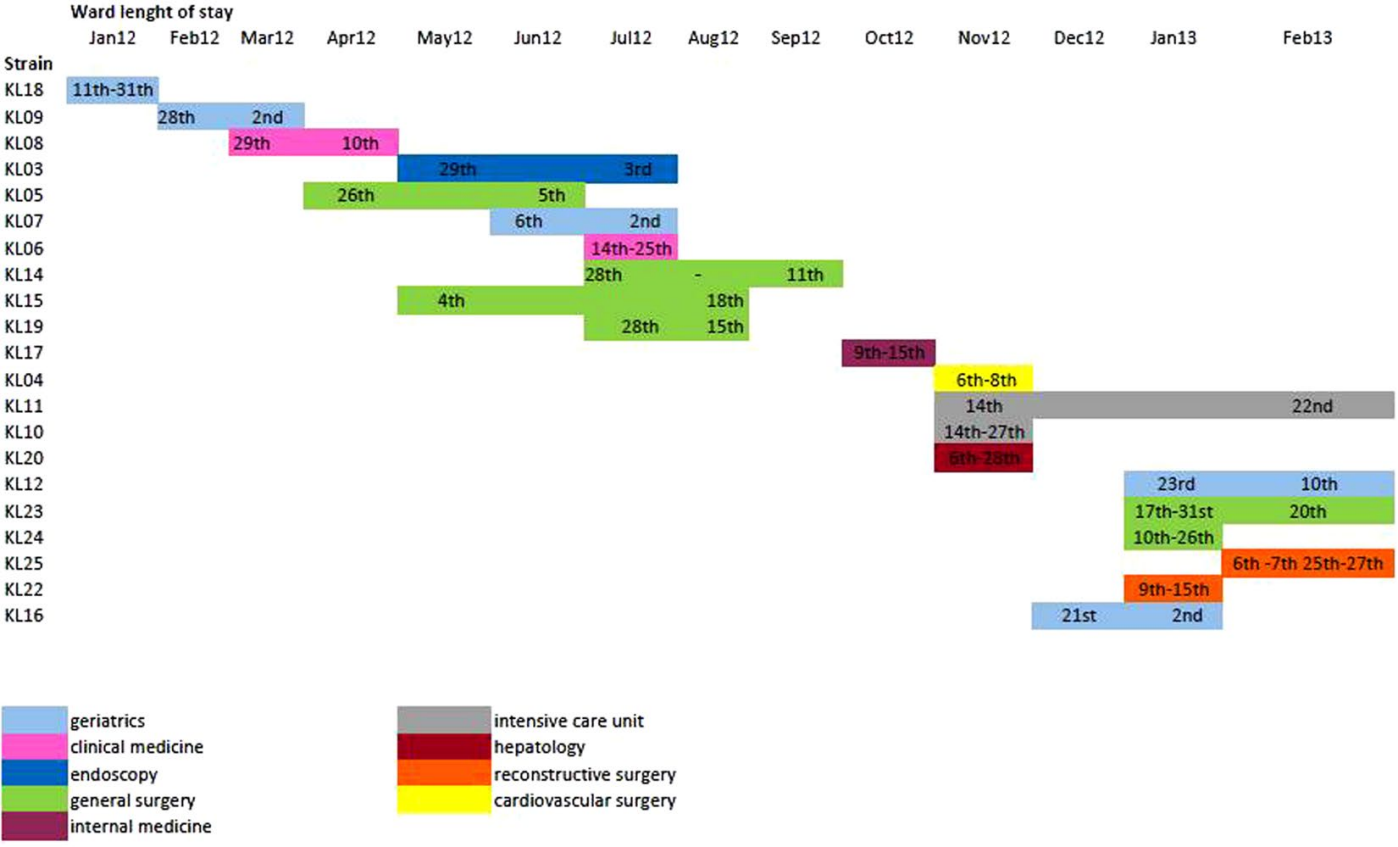
Timeline representing the *K*. *pneumoniae* MDR and KPC isolated in relationship with the ward and the length of stay in each ward. On the X-axis, ward length of stay dated by months and days, is reported. Different colours in the legend are to indicate the different hospital wards.

The epidemic curve based on the number of isolates collected during the study period (Fig. 1b) showed two discrete consecutive periods of infections (November 2012 and January 2013) separated by 46 days. A timeline representing the *K*. *pneumoniae* MDR and KPC isolated in relationship with the ward and the length of stay in each ward was built (Fig. 2). It showed that the general surgery was involved continuously between April and September 2012, whereas the geriatric ward was involved in three distinct periods separated by 60 and 120 days respectively (January-March 2012, June-July 2012 and December 2012-February 2013), as in Fig. 2.

### Antimicrobial susceptibility testing (AST) results

AST profile have been reported in the Supplementary Table S1. All *Klebsiella pneumoniae* strains resulted MDR and KPC. 12/21 (57%) strains were susceptible only to colistin, whereas 9/21 (43%) showed a more variable or complex AST profile and 3/9 (33%) strains were colistin resistant.

### Genomic and phylogenetic analysis

**Figure 3:**
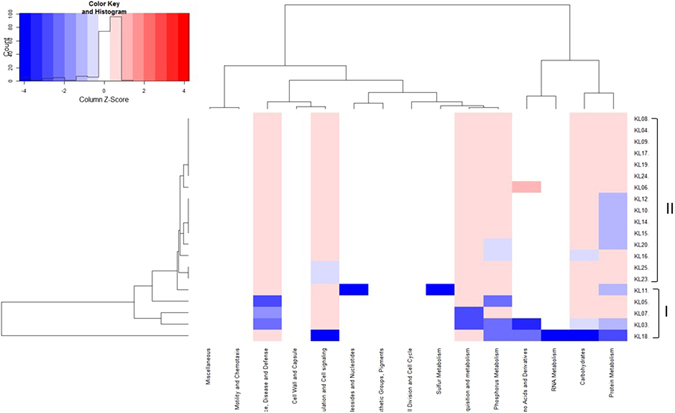
Genetic expression of *Klebsiella pneumoniae* isolates. Group I and II are highlighted. In the X-axis genes divided by group are represented. Blue boxes represent underexpressed gene, whereas red boxed overexpressed genes. The white color correspond to the “zero” value indicating absence of over/under gene expression.

*De novo* assemblies were annotated with RAST removing the genome of the strain KL22 because it showed a very different expression than the other strains. In Fig. 3, there were two different groups of expression: one group (group I) included KL03, KL05, KL07, KL11 and KL18; and the other one (group II) included the remaining sequences. Where the group I was under expressed the group II was over expressed, whereas for most of the gene groups all the sequences had a similar expression.

Annotations were confirmed using Prokka, the type of genes annotated are reported in Table S2. The recombination analysis (Supplementary Figure S1) showed little recombination due to the possible translocation between the strains. After removing SNPs introduced by recombination, phylogenetic signal was assessed using a transition/transversion vs. divergence graph and the Xia’s test (p < 0.001) that did not show evidence for substitution saturation (Supplementary Figure S2 and Supplementary Table S3). Likelihood mapping analysis reported 7.7% of star-like signal (phylogenetic noise (Supplementary Figure S3). This indicated that enough signal for phylogenetic inference existed.

Maximum Likelihood tree of *K*. *pneumoniae* core genome SNPs alignment has been reported in the Supplementary Figure S4. A statistically significant clade was highlighted (clade A). KL20 strain appeared to be more divergent than the other strains (as confirmed from the annotation analysis); indeed in the tree, this strain was considered as outgroup.

**Figure 4:**
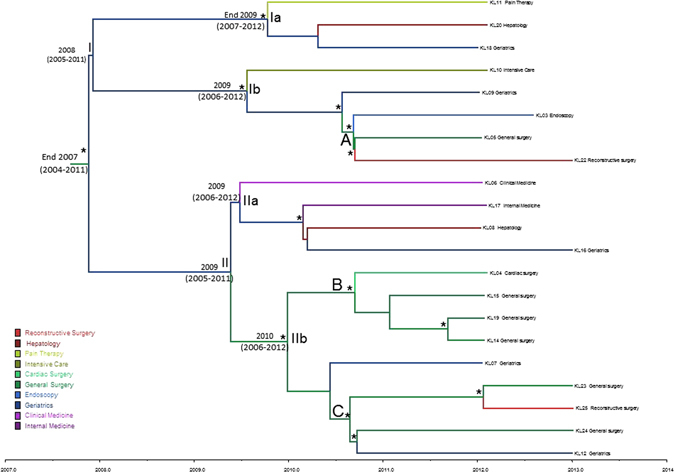
Maximum clade credibility (MCC) tree with Bayesian phylogeography recostruction of *Klebsiella pneumoniae* isolates. Branches are scaled in time and colored according to the legend to the left where each color represents the geographic location of the sampled sequence (tip branches), as well as of the ancestral lineage (internal Branches) inferred by Bayesian phylogeography. Significant posterior probability support (pp ≥ 0.9) as indicated by an asterisk. Clade and clusters are highlighted.

Figure 4 showed the phylogeographic analysis performed on the hqSNPs of the core genome to investigate the evolution of the *K*. *pneumoniae*. The exponential growth demographic model with a relaxed molecular clock was selected as the most appropriate to describe the evolutionary history of *Klebsiella pneumoniae*. Molecular clock calibration estimated the evolutionary rate of the *K*. *pneumoniae* SNPs core genome alignment at 4.97 × 10^−3^ substitutions site per year (95% HPD 9.98 × 10^−3^–9.67 × 10^−4^).

The date of the time of the most common recent ancestor (tMRCA) corresponded to the end of 2007 (HPD 95% 2004–2011) with the most probable location for the MRCA in the general surgery ward IIIW (Supplementary Figure S5). The maximum clade credibility (MCC) tree was composed by two major clades (clade I and II, dating back 2008 and 2009, respectively), both putatively originating in the geriatrics ward (hospital area IIIW). In Clade I two different clusters probably originating in the geriatric ward (hospital area IIIW), are evident. Cluster Ia dating to end of the year 2009 (HPD 95% 2007–2012) included sequences from pain therapy, hepatology and geriatrics ward. Cluster Ib dating to 2009 (HPD 95% 2006–2012) with sequences from intensive care, geriatrics, endoscopy, reconstructive surgery and general surgery wards.

The KL10 strain was isolated from a patient admitted to the intensive care ward, but three days prior to culture collection was transferred to the geriatrics ward. Interestingly, this cluster contained also a group of isolates (A) including three patients temporally and spatially clustered from general surgery (hospital area IIIW).

In Clade II, two different clusters were evidenced. Cluster IIa dated back 2009 (HPD 95% 2006–2012) and possible location was geriatrics ward (hospital area IIE); this cluster included sequences from clinical medicine, internal medicine, hepatology and geriatrics wards. Cluster IIb dated back 2010 (HPD 95% 2006–2012) with general surgery ward as possible location and IIE as hospital area. The KL23 sequence belonged to a patient admitted in general surgery ward, but in the six/five days before the culture request for suspected infection, he stayed in intensive care ward. The group labelled as B had as possible location general surgery ward in hospital area IIIE; it included sequences from general surgery and cardiac surgery wards sampled from August to November 2012. The KL14 and KL19 sequences belonged to two patients admitted both in general surgery ward in the similar period in two different hospital areas (IIIE and IIE respectively). These patients in the four days before the culture request for suspected infection, were in intensive care ward in the same night in two beds next each other (ICU bed 05 and ICU bed 03 respectively). The possible location of group labelled as C was general surgery ward in hospital area IIE; this cluster included sequences from general surgery, reconstructive surgery and geriatrics wards sampled in January and February 2013.

Based upon antimicrobial susceptibility testing results, 9/21 (43%) *Klebsiella pnemomiae* (KL05, KL03, KL22, KL16, KL10, KL20 and KL07) strains showed a more variable AST profile: strains KL05, KL03 and KL22 closely related within the cluster Ib, showed different phenotype than KL10 and KL09 belonging to the same cluster; strain KL07 was different from the other strains into the statistically supported group C, in agreement with the AST profile; strain KL20 appeared to be very close to strain KL18 without any statistically support in agreement with the different AST phenotype.

Three of nine (33%) strains (KL11, KL15 and KL06) were colistin resistant and interspersed in the tree each out of the proper subclade (Fig. 4).

At the MLST analysis, *K*. *pneumoniae* MDR and KPC strains resulted to be ST512 except one (KL05) ST650, different locus variants belonging to the same clonal complex (CC258).

**Figure 5:**
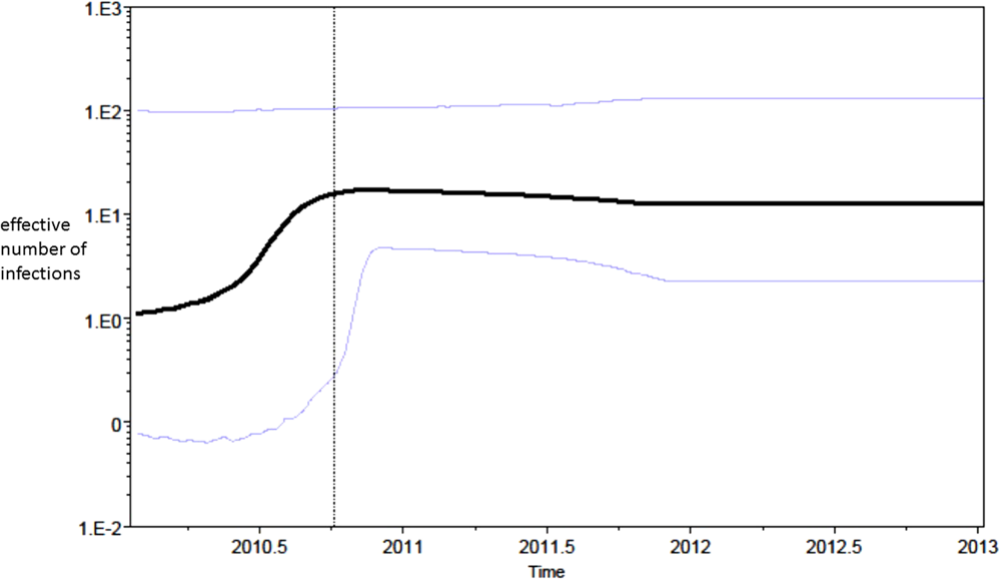
Bayesian skyline plot (BSP) of the *Klebsiella pneumoniae* isolates. The effective number of infections is reported on the Y-axis. Time is reported in the X-axis. The coloured lines correspond to the credibility interval based on 95% highest posterior density interval (HPD).

Figure 5 shows a Bayesian skyline plot for the effective population size of *Klebsiella pnemoniae* core genome SNPs alignment. Even if the median valued would suggest an exponential growth from 2010 to the end of the same year, the confidence intervals for that period are so wide that other growth scenarios (e.g. constant growth) cannot be excluded. In 2011, a light decrease followed by a plateau until 2013 started, moreover the Bayesian analysis reported R_0_ of 1.573 (95% confidence interval from 0.763 to 2.59).

## Discussion

In this study, the combination of epidemiological analysis and high-resolution whole-genome sequencing has been shown to be valuable for nosocomial outbreaks investigation. Next generation sequencing (NGS) is becoming an important framework for clinical diagnostics. The NGS methodology has been recently used to characterize pathogens in different contexts^13–21^, moreover the reasonable cost of the analysis make it the possibility to use also in a routinary diagnostic setting^22^ and may be an important resource for nosocomial bacterial surveillance^23^. The study was designed providing a “snap shot” of K*lebsiella pneumoniae* in hospital setting combining data for surveillance with molecular ones. Moreover, Bayesian phylogenetic and phylogeographic analyses have been powerful tools used to follow the spread of *Klebsiella pneumoniae* in different wards and different time.

The epidemic curve based on the number of isolates collected during the study period (Fig. 1b) showed two different picks of infections (November 2012 and January 2013) separated by 46 days. This could indicate a single outbreak period (Nov 2012 to Jan 2013) with unobserved cases in December, making the investigators aware that an improvement in preventive measures need to be adopted.

The Bayesian tree dated the TMRCA indicating that strains isolated between 2012 and 2013 could be introduced in the hospital setting since the end of the year 2007 with the most probable location in general surgery ward. The spread of the pathogen strains across different wards and floors of the hospital could suggest that the infections could be probably transmitted by staff members (medical or paramedical) who have free access to all wards of the hospital, than patients or their relatives. Furthermore, It seems possible to assume that the general surgery ward could play an important role in disseminating the bacteria across the hospital when patients return to their ward after a surgical procedure.

Moreover, the phylogenetic tree showed two different epidemic entrance in the hospital (2008 and 2009 years). Connecting epidemiological data with phylogenetic analysis was evident as ERCP performed in four patients were found in two different groups A and B. Interestingly, within group A, strain KL09 isolated in a patient coming from a different hospital was admitted with documented infection in March 2012. The phylogenetic tree showed how this strain is a sort of outgroup that probably infected the other components of group A. In this group are included strains KL05 and KL03 from patients submitted to ERCP suggesting that after its introduction the strain circulation was maintained through the nosocomial endoscopic and post-surgery dressing procedures. Strain KL22 even if not receiving ERCP treatment or not admitted in the same ward it is clearly significantly related with the other three strains in group A probably for surgery or visit room sharing.

In group B, isolates KL14 and 19 were from patients admitted both in general surgery, in overlapping periods, but in two different hospital areas (IIIE and IIE respectively). These patients in the four days before the culture request were in ICU in the same night in two beds next each other (ICU bed 05 and ICU bed 03 respectively). In these last two patients it was probable the man-to-man transmission as evident by the phylogenetic tree and confirmed by the epidemiological data. Anyway, we cannot exclude if fomites, or devices, or persons encountering these patients have been a sort of “bridge” to transmit the infection between them.

Phylogenetic analysis also revealed the presence of another statistically supported group (group C). In this group strains KL23 and KL25 were from patients that in overlapping period were admitted in different ward at the same floor but in different hospital areas (II East and II West). The tree topology showed a significant relationship between them suggesting that the strain could be the same probably acquired during the hospital stay. Another statistically supported cluster of group C was evident in the tree for strains KL12 and KL24. These isolates were from patients admitted in the same floor but in different hospital areas (II West and II East, respectively). In this case, the classical epidemiology does not help to clarify the way of transmission, whereas the phylogenetic analysis clearly suggest the same strain infecting these two patients. It is noteworthy, that patients of group C were in overlapping period hospitalized in the same floor, the second floor.

At a first sight, the Bayesian skyline could suggest in part an exponential growth of *K*. *pneumoniae* infections from 2010 reaching a plateau in 2013, but considering the confidence intervals for the same period the constant growth scenario cannot be excluded. Exponential as well as constant growth are in line with the absence of microbiological surveillance at hospital admission looking for multidrug-resistant (MDR) bacteria until the year 2013.

By reconstructing the demographic of bacterial population, it was also possible to estimate the R_0_ value for *Klebsiella pneumoniae* infections. Even if the colonization state, during which bacteria can transiently or persistently colonize an individual, can represent a bias for R_0_ values estimate, the R_0_ calculated for our strains, ranging from 0.763 to 2.59, does not suggest a clear indication that the outbreak is self-sustaining over the studied period being the lower limit range value <1.

In addition to the preventive measures realized since 2013, consisting in patient contact isolation upon MDR bacteria detection, some others measure should be improved based on the results of the present study. Microbiological surveillance of rectal, nasal and pharyngeal swabs could be included for inpatients as well as outpatients referred to endoscopic procedures such as ECRP. Staff hand hygiene adhesion should be improved by collection of data of adherence and continuing education program; the microbiological surveillance should be extended to operative endoscope used in ERCP procedures by microbiological sampling of different parts and channel of the instrument to check the efficiency of the sterilization procedures.

In conclusion, our study showed the complex transmission and circulation dynamics of nosocomial strains. The cross-talk between classical and molecular epidemiology, when both are known, allowed us to accurately define the way of strains transmission. The molecular epidemiology based on phylogenetic analysis could represent a useful tool to support the classical epidemiology in the MDR pathogen surveillance. The two different approaches if adopted together could aid to trace exactly the way of transmission of the pathogen and perform a focused action plan in the wards were the transmission began.

## Methods

### Sample collection and epidemiologic investigation

Between January 2012 and February 2013, twenty-one *Klebsiella pneumoniae* MDR and KPC strains were collected from inpatients of the tertiary care 280-bed University Hospital Campus Bio-Medico of Rome, Italy. Patients were admitted in different wards distributed along four floors and two distinct hospital locations (East and West area) (Supplementary Figure S5).

Clinical samples were obtained from patients with signs of bacterial infections as part of routine clinical evaluation as assessed by the clinical team. *Klebsiella pneumoniae* strains were obtained from routine processing of clinical samples used for bacterial infection diagnosis. All methods were carried out in accordance with relevant guidelines and regulations in effect at the University Hospital Campus Bio-Medico of Rome.

The study was approved by the local Ethical Committee of the Univesrity Campus Bio-Medico of Rome (prot. 48/16 OSS ComEt CBM). Informed consent was obtained from all subjects at ward admission.

*Klebsiella pneumoniae* isolates were identified by MALDI-TOF using the MALDI Biotyper 3.0 software version (Bruker Daltonics, GmbH, Bremen, Germany)^21^. *Klebsiella pneumoniae* antimicrobial susceptibility tests were performed by Vitek2 Compact (bioMérieux, Marcy l’Etoile, France) and the resistant phenotype further confirmed with the Kirby-Bauer method according to Clinical Laboratory Standard Institute (CLSI) and European Committee for Antimicrobial Susceptibility Test (EUCAST)^24^. The Hospital Infection Control Team and the investigators of the Clinical Pathology and Microbiology Unit performed a detailed epidemiologic investigation. Medical records were examined for epidemiologic data collection, such as dates of patients ward admission and discharge, bed assignment, diagnostic and therapeutic procedures and microbiological laboratory results.

Two epidemic curves based one on the number of *Klebsiella pneumoniae* isolated in the temporal frame of the study and one on the number of isolates for each ward have been developed.

### Whole-genome sequencing (WGS)

Bacterial DNA was extracted by the EZ1 DNA tissue kit (Qiagen, Dusseldorf, Germany) and whole genome sequenced by Next Generation Sequencing using Illumina MiSeq II sequencer (Library Preparation Kit: Nextera XT DNA Sample Prep Kit, Indexing: Dual Indexing Reagent Kits: MiSeq Reagent Kit v3, Analysis Workflow: Resequencing, Analysis Software: MiSeq Reporter). Sequencing reads from the isolates obtained in this study were assembled using an established bioinformatics pipeline as previously described^25^. *De novo* assemblies were constructed using the SPAdes^26^, and contigs ordered using the strain CAV1596 as reference sequence with Mauve v. 2.3.1^27, 28^. The strain CAV1596 was selected as the best reference genome performing an analysis with PARSNP^29^ and visualizing the output with Gingr^29^ to identify the more appropriate reference genome. Gingr provides an interactive display of multi-alignments, variants and the phylogenetic tree estimated from the core genome alignment.

Sequences were then aligned with Progressive Mauve^30^.

*De novo* assemblies were annotated with RAST (http://rast.nmpdr.org/), a fully automated service generating high-quality annotations for complete or nearly complete bacterial and archaeal genomes, and used to generate a heat-map of gene expression^31^. Annotations were confirmed using Prokka, which relies on external feature prediction tools to identify the coordinates of genomic features within contigs^32^.

The pangenome was then assessed to determine the core genome, which typically includes housekeeping genes for cell envelope or regulatory functions^33^.

Pangenome analyses was performed using Roary^34^. Recombination was evaluated using Gubbins (Genealogies Unbiased By recomBinations In Nucleotide Sequences)^35^.

Single-nucleotide polymorphisms (SNPs) were based on the core genome shared by all isolates. SNPs were extracted as variable sites using MEGA 6^36^ removing all ambiguous sites and gaps.

### Phylogenetic and phylogeographic analyses

Phylogenetic signal was assessed by likelihood mapping using TreePuzzle^37^. A transitions/transversions vs. divergence graph as well as the Xia’s test of substitution saturation were implemented in DAMBE^38^.

The HKY+I+G nucleotide substitution model was chosen as best-fitting model by using the hierarchical likelihood ratio test (Modeltest, implemented in PAUP*4). Statistical support for internal branches of the Maximum Likelihood (ML) tree was evaluated by bootstrapping (1000 replicates) and fast likelihood-based sh-like probability (SH-aLRT). ML analysis was performed with IQ-TREE^39^ and visualized in FigTree 1.4.0.

The evolutionary rate was estimated by calibrating a molecular clock using known sequences sampling times with the Bayesian Markov Chain Monte Carlo (MCMC) method implemented in BEAST v. 1.8.2 (http://beast.bio.ed.ac.uk)^40, 41^. In order to investigate the demographic history, independent MCMC runs were carried out enforcing both a strict and relaxed clock with an uncorrelated log normal rate distribution and one of the following coalescent priors: constant population size, exponential growth, non-parametric smooth skyride plot Gaussian Markov Random Field (GMRF), and non-parametric Bayesian skyline plot (BSP)^40, 42, 43^ with ascertainment bias correction. Marginal likelihoods estimates for each demographic model were obtained using path sampling and stepping stone analyses^44–46^. Uncertainty in the estimates was indicated by 95% highest posterior density (95% HPD) intervals, and the best fitting model for each data set was by calculating the Bayes Factors (BF)^44, 47^. In practice, any two models can be compared to evaluate the strength of evidence against the null hypothesis (*H*_*0*_), defined as the one with the lower marginal likelihood: 2*ln*BF <2 indicates no evidence against *H*_*0*_; 2–6, weak evidence; 6–10: strong evidence, and >10 very strong evidence. Chains were conducted for at least 100 × 10^6^ generations, and sampled every 10000 steps for each molecular clock model. Convergence of the MCMC was assessed by calculating the ESS for each parameter. Only parameter estimates with ESS’s of >250 were accepted. The maximum clade credibility (MCC) tree was obtained from the trees posterior distributions, after a 10% burn-in, with the Tree-Annotator software v 1.8.2, included in the Beast package^40, 41^. Statistical support for specific monophyletic clades was assessed by calculating the posterior probability (pp > 0.90).

The continuous-time Markov Chain (CTMC) process over discrete sampling locations implemented in BEAST^41^ was used for the phylogeography inference, by using the Bayesian Stochastic Search Variable Selection (BSSVS) model, which allows the diffusion rates to be zero with a positive prior probability. Locations considered were the different wards of the hospital and different hospital areas. Comparison of the posterior and prior probabilities of the individual rates being zero provided a formal BF for testing the significance of the linkage between locations. The MCC tree with the phylogeographic reconstruction was selected from the posterior tree distribution after a 10% burn-in using the Tree Annotator.

An epidemic curve based on the number of isolates falling within each phylogenetic clade during the study period has been developed.

### R_0_ estimate

Using BEAST, the basic reproduction number (R_0_) was calculated for core genome SNPs alignment under a relaxed clock with Birth-Death Basic Reproductive Number demographic model^48^. R_0_ is the basic reproductive number (infectivity) of a pathogen, i.e. the average number of secondary infections caused by each primary infected individual. In a pathogen population exponentially growing at rate r, where D is the average duration of infectiousness, it can be shown that if the pathogen is transmitted at the same rate during the total length of infection, then R_0_ = rD + 1^49^.

### Multilocus Sequence typing (MLST)

MLST was performed according to the protocol described by Diancourt and colleagues^50^ based on seven housekeeping genes: *gapA* (glyceraldehyde 3-phosphate dehydrogenase), *infB* (translation initiation factor 2), *mdh* (malate dehydrogenase), *pgi* (phosphoglucose isomerase), *phoE* (phosphorine E), *rpoB* (betasubunit of RNA polymerase) and *tonB* (periplasmic energy transducer). The MLST database used for *K*. *pneumoniae* is available at http://www.pasteur.fr.

## Electronic supplementary material

Supplementary figures and Tables
