# Supplementary material for: Multi-drug resistant Klebsiella pneumoniae strains circulating in hospital setting: whole-genome sequencing and Bayesian phylogenetic analysis for outbreak investigations
Source: Sci Rep. 2017 Jun 14;7:3534. doi: 10.1038/s41598-017-03581-4 (PMC5471223; doi:10.1038/s41598-017-03581-4)

Multi-drug resistant *Klebsiella pneumoniae* strains circulating in hospital setting: whole-genome sequencing and Bayesian phylogenetic analysis for outbreak investigations

Eleonora Cella[1,2*], Massimo Ciccozzi[1,3*], Alessandra Lo Presti[1], Marta Fogolari[3], Taj Azarian[4], Mattia Prosperi[5], Marco Salemi[6], Michele Equestre[7], Francesca Antonelli[3], Alessia Conti[3], Marina De Cesaris[3], Silvia Spoto[8], Raffaele Antonelli Incalzi[9], Roberto Coppola[10], Giordano Dicuonzo[3], Silvia Angeletti[3]

*These authors contributed equally to this work.

[1]Department of Infectious, Parasitic and Immune-Mediated Diseases, Istituto Superiore di Sanità, Rome, Italy.

[2]Department of Public Health and Infectious Diseases, Sapienza University of Rome, Rome, Italy

[3]Unit of Clinical Pathology and Microbiology, University Campus Bio-Medico of Rome, Italy.

[4]Department of Epidemiology, Center for Communicable Disease Dynamics. Harvard's T.H. Chan School of Public Health, Boston MA.

[5]Department of Epidemiology, University of Florida, Gainesville, FL, USA.

[6]Department of Pathology, Immunology, and Laboratory Medicine, Emerging Pathogens Institute, University of Florida, Gainesville, FL, USA.

[7]Department of Cell Biology and Neurosciences, Istituto Superiore di Sanità, Rome, Italy.

[8]Internal Medicine Department, University Hospital Campus Bio-Medico, Rome, Italy.

[9]Unit of Geriatrics, Department of Medicine, University Campus Bio-Medico of Rome, Italy.

[10]Department of Surgery, University Campus Bio-Medico of Rome, Italy.

Corresponding author: Massimo Ciccozzi

Department of Infectious Parasitic and Immunomediated Diseases, Reference Centre on Phylogeny, Molecular Epidemiology and Microbial Evolution (FEMEM)/ Epidemiology Unit, National Institute of Health, Rome, Italy.

Condensed Title: Multi-drug resistant Klebsiella pneumoniae strains circulating in hospital setting

Supplementary Table S1. *Klebsiella pneumoniae* isolates Antimicrobial susceptibility test (AST)

**Isolates**	**Isolation date**	**AST**	**ST**
KL18	Jan 2012	Piperacillin/tazobactam >128 (R)<br>Meropenem >8 (R)<br>Imipenem >16 (R)<br>Gentamicin 4 (I)<br>Amikacin >16 (R)<br>Ciprofloxacin >4 (R)<br>Trimethoprim/sulfamethoxazole >4/76 (R)<br>Colistin <1 (S)	512
KL09	Mar 2012	Piperacillin/tazobactam >128 (R)<br>Meropenem >8 (R)<br>Imipenem >16 (R)<br>Gentamicin 4 (I)<br>Amikacin >16 (R)<br>Ciprofloxacin >4 (R)<br>Trimethoprim/sulfamethoxazole >4/76 (R)<br>Colistin <1 (S)	512
KL08	Apr 2012	Piperacillin/tazobactam >128 (R)<br>Meropenem >8 (R)<br>Imipenem >16 (R)<br>Gentamicin 4 (I)<br>Amikacin >16 (R)<br>Ciprofloxacin >4 (R)<br>Trimethoprim/sulfamethoxazole >4/76 (R)<br>Colistin <1 (S)	512
KL14	Aug 2012	Piperacillin/tazobactam >128 (R)<br>Meropenem >8 (R)<br>Imipenem >8 (R)<br>Gentamicin 4 (I)<br>Amikacin >16 (R)<br>Ciprofloxacin >4 (R)<br>Trimethoprim/sulfamethoxazole >4/76 (R)<br>Colistin <1 (S)	512
KL19	Aug 2012	Piperacillin/tazobactam >128 (R)<br>Meropenem >8 (R)<br>Imipenem >8 (R)<br>Gentamicin 4 (I)<br>Amikacin >16 (R)<br>Ciprofloxacin >4 (R)<br>Trimethoprim/sulfamethoxazole >4/76 (R)	512

		Colistin <1 (S)	
KL17	Oct 2012	Piperacillin/tazobactam >128 (R)<br>Meropenem >8 (R)<br>Imipenem >16 (R)<br>Gentamicin 4 (I)<br>Amikacin >16 (R)<br>Ciprofloxacin >4 (R)<br>Trimethoprim/sulfamethoxazole >4/76 (R)<br>Colistin <1 (S)	512
KL05	May 2012	Piperacillin/tazobactam >128 (R)<br>Meropenem >8 (R)<br>Imipenem >8 (R)<br>Gentamicin >4 (R)<br>Amikacin >16 (R)<br>Ciprofloxacin >4 (R)<br>Trimethoprim/sulfamethoxazole >4/76 (R)<br>Colistin <1 (S)	**650**
KL03	Jun 2012	Piperacillin/tazobactam >128 (R)<br>Meropenem >8 (R)<br>Imipenem >8 (R)<br>Gentamicin 4 (I)<br>Amikacin >16 (R)<br>Ciprofloxacin >4 (R)<br>Trimethoprim/sulfamethoxazole <1/19 (S)<br>Colistin <1 (S)	512
KL06	Jul 2012	Piperacillin/tazobactam >128 (R)<br>Meropenem >8 (R)<br>Imipenem >8 (R)<br>Gentamicin >4 (R)<br>Amikacin >16 (R)<br>Ciprofloxacin >4 (R)<br>Trimethoprim/sulfamethoxazole >4/76 (R)<br>Colistin >16 (R)	512
KL20	Nov 2012	Piperacillin/tazobactam >128 (R)<br>Meropenem >16(R)<br>Imipenem >8 (R)<br>Gentamicin <1 (S)<br>Amikacin<2 (S)<br>Ciprofloxacin >4 (R)<br>Trimethoprim/sulfamethoxazole >4/76 (R)<br>Colistin <1 (S)	512
KL10	Nov 2012	Piperacillin/tazobactam >128 (R)<br>Meropenem >16(R)<br>Imipenem >8 (R)<br>Gentamicin <1 (S)<br>Amikacin<2 (S)<br>Ciprofloxacin >4 (R)	512

		Trimethoprim/sulfamethoxazole >4/76 (R)<br>Colistin <1 (S)	
KL16	Jan 2013	Piperacillin/tazobactam >128 (R)<br>Meropenem >16(R)<br>Imipenem >8 (R)<br>Gentamicin <1 (S)<br>Amikacin>64 (R)<br>Ciprofloxacin >4 (R)<br>Trimethoprim/sulfamethoxazole >4/76 (R)<br>Colistin <1 (S)	512
KL22	Jan 2013	Piperacillin/tazobactam >128 (R)<br>Meropenem >8 (R)<br>Imipenem >8 (R)<br>Gentamicin 4 (I)<br>Amikacin >16 (R)<br>Ciprofloxacin >4 (R)<br>Trimethoprim/sulfamethoxazole >4/76 (R)<br>Colistin <1 (S)	512
KL12	Jan 2013	Piperacillin/tazobactam >128 (R)<br>Meropenem >8 (R)<br>Imipenem >8 (R)<br>Gentamicin 4 (I)<br>Amikacin >16 (R)<br>Ciprofloxacin >4 (R)<br>Trimethoprim/sulfamethoxazole >4/76 (R)<br>Colistin <1 (S)	512
KL23	Jan 2013	Piperacillin/tazobactam >128 (R)<br>Meropenem >8 (R)<br>Imipenem >8 (R)<br>Gentamicin 4 (I)<br>Amikacin >16 (R)<br>Ciprofloxacin >4 (R)<br>Trimethoprim/sulfamethoxazole >4/76 (R)<br>Colistin <1 (S)	512
KL24	Feb 2013	Piperacillin/tazobactam >128 (R)<br>Meropenem >8 (R)<br>Imipenem >8 (R)<br>Gentamicin 4 (I)<br>Amikacin >16 (R)<br>Ciprofloxacin >4 (R)<br>Trimethoprim/sulfamethoxazole >4/76 (R)<br>Colistin <1 (S)	512
KL25	Feb 2013	Piperacillin/tazobactam >128 (R)<br>Meropenem >8 (R)<br>Imipenem >8 (R)<br>Gentamicin 4 (I)<br>Amikacin >16 (R)	512

		Ciprofloxacin >4 (R)<br>Trimethoprim/sulfamethoxazole >4/76 (R)<br>Colistin <1 (S)	
KL04	Nov 2012	Piperacillin/tazobactam >128 (R)<br>Meropenem >8 (R)<br>Imipenem >8 (R)<br>Gentamicin 4 (I)<br>Amikacin >16 (R)<br>Ciprofloxacin >4 (R)<br>Trimethoprim/sulfamethoxazole >4/76 (R)<br>Colistin <1 (S)	512
KL15	Aug 2012	Piperacillin/tazobactam >128 (R)<br>Meropenem >8 (R)<br>Imipenem >8 (R)<br>Gentamicin 4 (I)<br>Amikacin >16 (R)<br>Ciprofloxacin >4 (R)<br>Trimethoprim/sulfamethoxazole >4/76 (R)<br>Colistin >16 (R)	512
KL11	Nov 2012	Piperacillin/tazobactam >128 (R)<br>Meropenem >8 (R)<br>Imipenem >8 (R)<br>Gentamicin 4 (I)<br>Amikacin >16 (R)<br>Ciprofloxacin >4 (R)<br>Trimethoprim/sulfamethoxazole <20 (S)<br>Colistin >16 (R)	512
KL07	Jun 2012	Piperacillin/tazobactam >128 (R)<br>Meropenem >8 (R)<br>Imipenem >8 (R)<br>Gentamicin 4 (I)<br>Amikacin >16 (R)<br>Ciprofloxacin >4 (R)<br>Trimethoprim/sulfamethoxazole <20 (S)<br>Colistin <1 (S)	512

Supplementary Table S2. Annotation of the genomes with Prokka.

Core genes (99% <= strains <= 100%)	2686
Soft core genes (95% <= strains < 99%)	1126
Shell genes (15% <= strains < 95%)	1670
Cloud genes (0% <= strains < 15%)	587
**Total genes**	6069

Supplementary Table S3. Xia's test for substitution saturation: testing whether the observed Iss is significantly lower than Iss.c in the *Klebsiella pneumoniae* core genome SNP alignment. IssSym is Iss.c assuming a symmetrical topology. IssAsym is Iss.c assuming an asymmetrical topology.

Iss	Iss.cSym	T	DF	P	Iss.cAsym	T	DF	P
0,17	0,74	75,91	631	0,00	0,49	42,9	631	0,00

Note: two-tailed tests are used. Interpretation of results: Iss < Iss.c & significant difference = little saturation; Iss > Iss.c & significant difference = useless sequences; Iss < Iss.c & non-significant difference = substantial saturation; Iss > Iss.c & non-significant difference = very poor for phylogenetics.

Supplementary Figure S1. Recombination analysis performed with Gubbins on *Klebsiella pneumoniae* core genome alignment. For each isolate, blocks representing the regions identified as recombinant are indicated by coloured blocks. Blue blocks are unique to a single isolate while red blocks are shared by multiple isolates through common descent. The horizontal position of the blocks represents their position in the alignment.

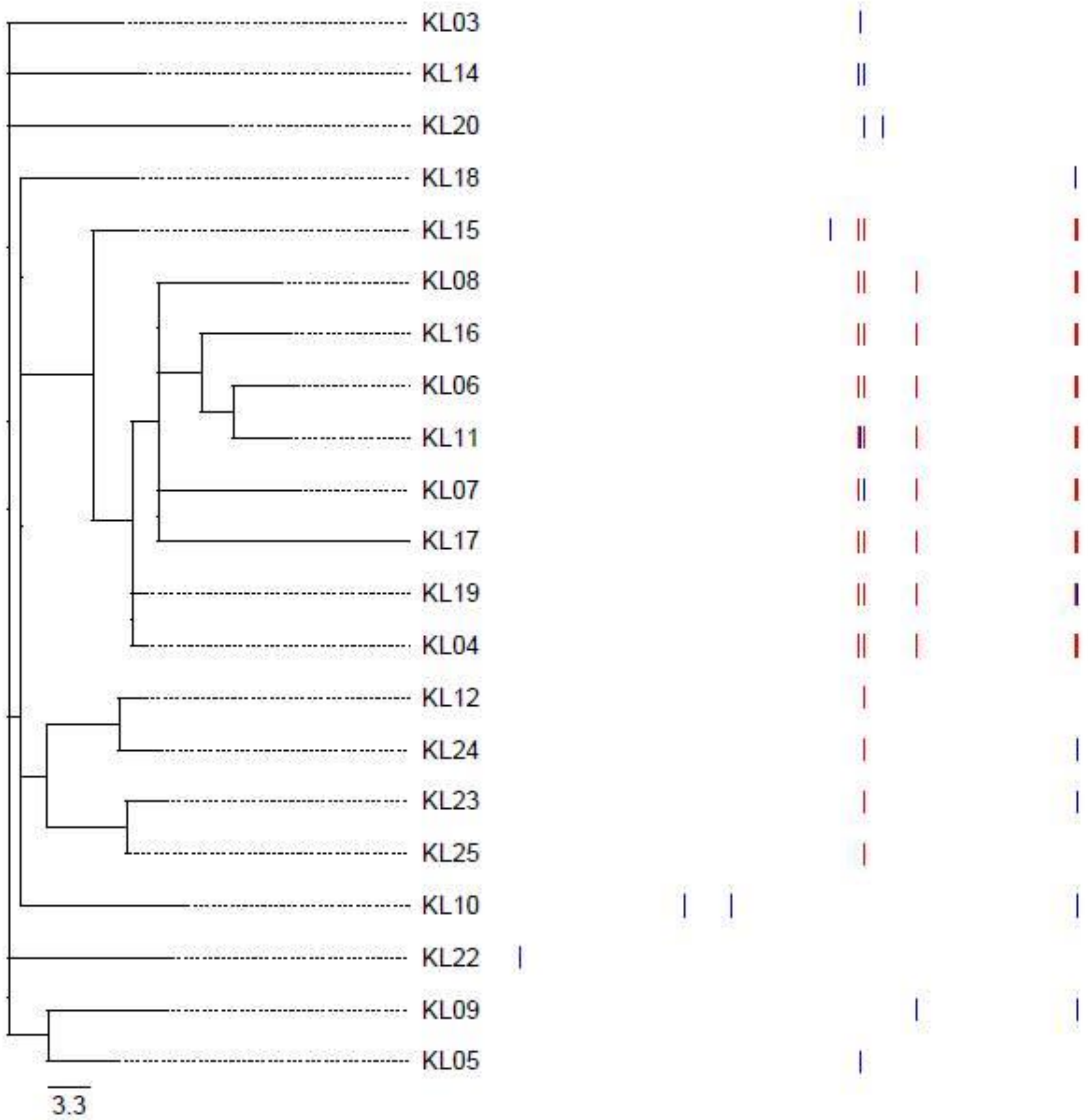

Supplementary Figure S2. Transitions/transversions vs. divergence graph for the *Klebsiella pneumoniae* core genome SNPs dataset.

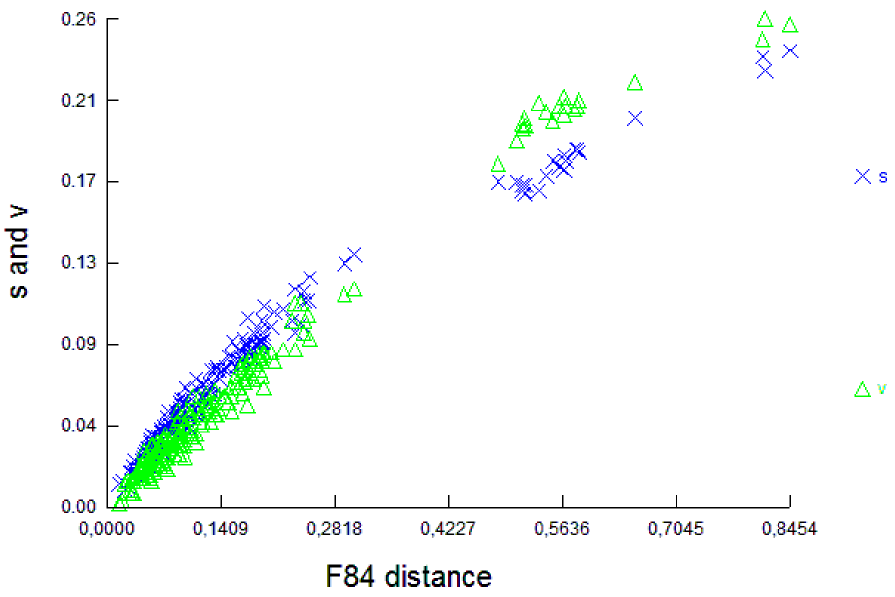

Supplementary Figure S3. Likelihood mapping of the *Klebsiella pneumoniae* core genome SNPs dataset. The dots inside the triangles represents the likelihood probabilities of the possible unrooted topologies for each quartet. Numbers indicate the percentage of dots in the centre of the triangle corresponding to phylogenetic noise (star-like trees).

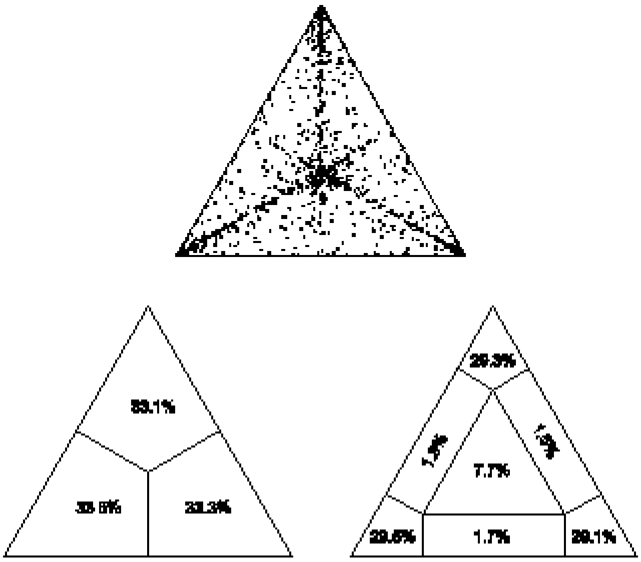

Supplementary Figure S4. ML of Klebsiella pneumoniae core genome SNPs alignment. * along the branches indicating a statistical value from bootstrap or sh-lrt analyses; ** indicating a statistical value from bootstrap and sh-lrt analyses.

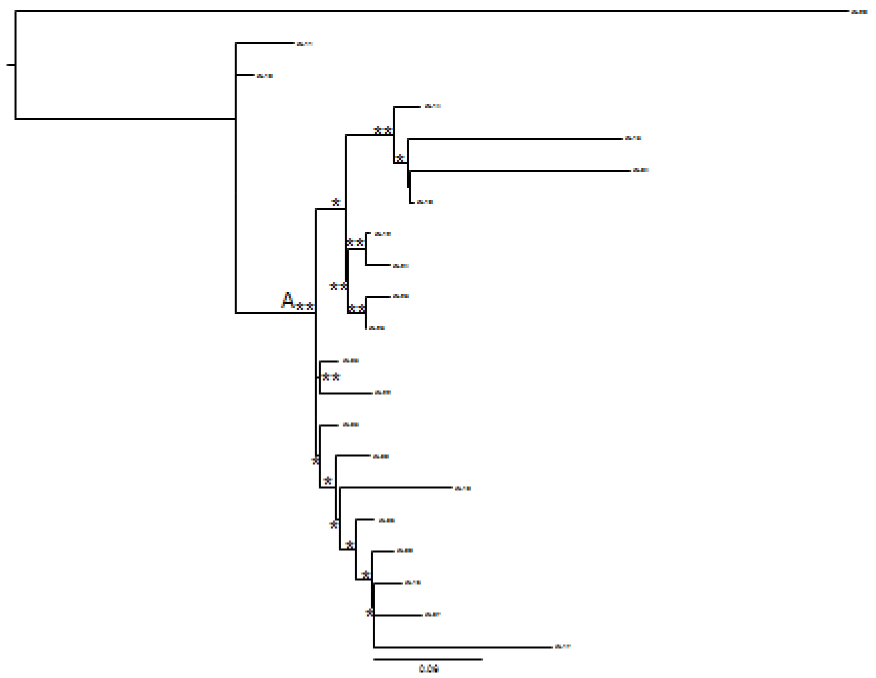

Supplementary Figure S5. Wards Layout of University Hospital Campus Bio-Medico.

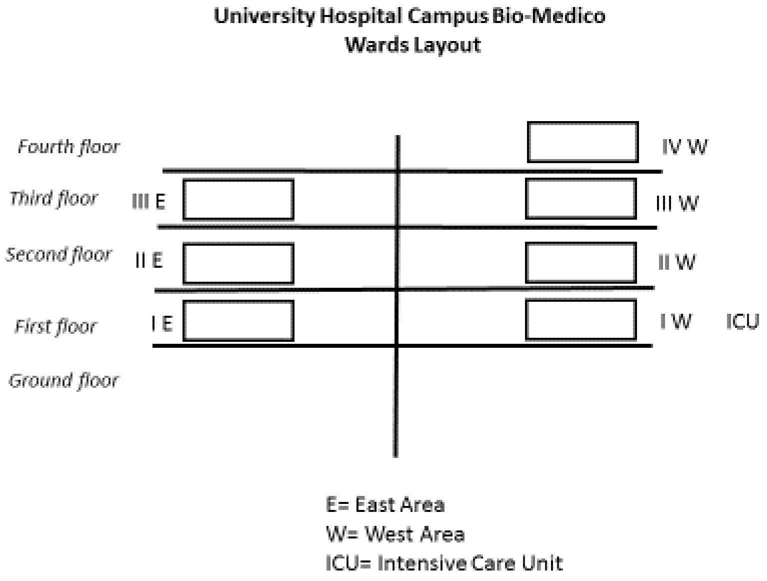

Supplement: Supplementary file 1 — Supplementary figures and Tables [file 41598_2017_3581_MOESM1_ESM.pdf]
